# Lipoproteins and Exosomes as Novel Carriers of Soluble Fms-Like Tyrosine Kinase-1 and Placental Growth Factor During Pregnancy

**DOI:** 10.1161/HYPERTENSIONAHA.124.23399

**Published:** 2024-07-30

**Authors:** Lunbo Tan, Martijn H. van Heugten, Ans C.M. Kluivers, Leonie van Vark-van der Zee, Monique T. Mulder, Xifeng Lu, A.H. Jan Danser, Koen Verdonk

**Affiliations:** Division of Vascular Medicine and Pharmacology, Department of Internal Medicine (L.T., A.C.M.K., L.v.V.-v.d.Z., M.T.M., A.H.J.D., K.V.).; Nephrology and transplantation, Department of Internal Medicine (M.H.H.), Erasmus MC, Rotterdam, The Netherlands.; Department of Obstetrics and Gynaecology (A.C.M.K.), Erasmus MC, Rotterdam, The Netherlands.; Women and Children’s Hospital of Chongqing Medical University, China (L.T.).; Clinical Research Center, The First Affiliated Hospital of Shantou University Medical College, China (L.T., X.L.).

**Keywords:** blood component removal, exosomes, lipoproteins, placenta growth factor, pre-eclampsia

Preeclampsia, complicating around 6% of pregnancies, has long-term consequences for the cardiovascular health of both mother and child.^1^ An angiogenic imbalance distinguishes the clinical manifestations of preeclampsia. It is reflected by an increase in the antiangiogenic factor sFlt-1 (soluble Fms-like tyrosine kinase-1) and a decrease in the free levels of the proangiogenic factor PlGF (placental growth factor), together resulting in an increased [sFlt-1]/[free PlGF] ratio.^1^ The latter is due, at least in part, to PlGF binding by sFlt-1.^2^ Consequently, reducing sFlt-1 has been proposed as a potential treatment approach for preeclampsia,^2^ for example, with LDL (low-density lipoprotein) apheresis.^3^ Simultaneously, exosome isolation via density-gradient ultracentrifugation has identified sFlt-1 and PlGF in extracellular vesicles.^4^ This observation raises the question whether it is the lipoprotein-containing fractions or exosomes that affect the interaction between sFlt-1 and PlGF. Since neither apheresis nor ultracentrifugation can distinguish between these particles, in the present study we investigated in which of the 2 circulating sFlt-1 and PlGF reside to better understand how to restore the angiogenic imbalance in preeclampsia.

Fast-protein liquid chromatography (FPLC) was applied to serum samples of 6 pregnant women not having preeclampsia (control) and 11 having preeclampsia (gestational age 30±5 and 33±4 weeks, respectively). All women had participated in a prospective cohort study evaluating the predictive role of the sFlt-1/PlGF ratio.^1^ No cholesterol differences were found in either the CM (chylomicron), VLDL (very-low-density lipoprotein), IDL (intermediate-density lipoprotein), LDL, HDL2 (high-density lipoprotein 2), HDL3, or free protein-containing fractions between the 2 groups (Figure [A]). In control women, sFlt-1 peaked in the LDL-containing fraction, with lower levels in the CM-containing fraction and the free protein-containing fraction and negligible amounts in the HDL2-, HDL3-, IDL-, and VLDL-containing fractions (Figure [A]). In preeclamptic women, sFlt-1 increased significantly in the HDL2- and HDL3-containing fractions (Figure [A]). Free PlGF occurred in the LDL-containing and HDL3-containing fractions in control women, with only the latter remaining in preeclampsia (Figure [A]). Total (=free+receptor-bound) PlGF, obtained after heating,^2^ occurred in all lipoprotein-containing fractions (peaking in LDL, HDL2, and HDL3), and this was identical in both groups (Figure [A]). Applying FPLC to recombinant sFlt-1 and PlGF in PBS resulted in their occurrence in the free protein-containing fraction only (data not shown). These data indicate that, in blood plasma, PlGF occurs exclusively in either exosomes or bound to lipoproteins, while sFlt-1 also occurs in these compartments and is unbound.

**Figure.**
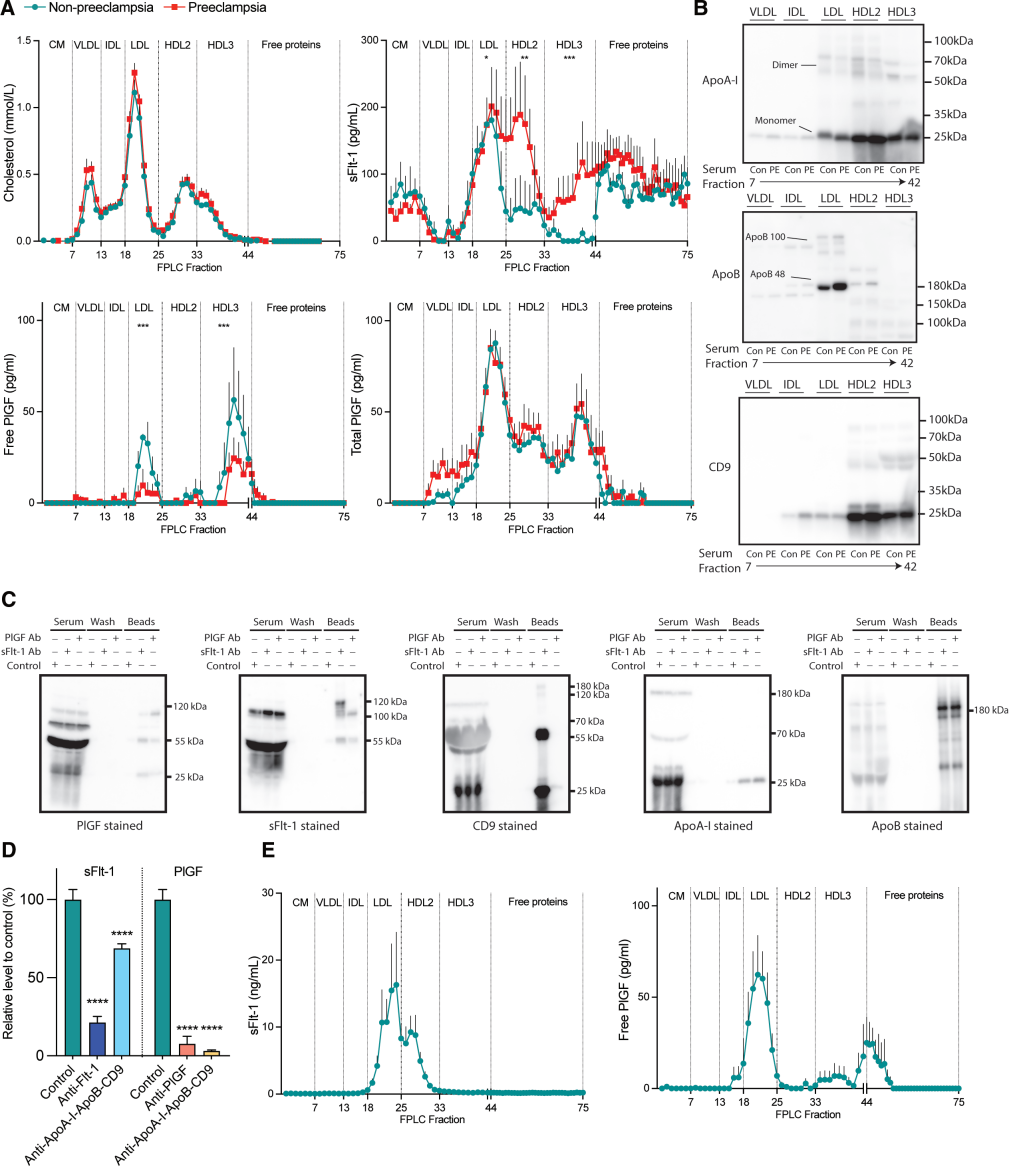
**The interaction of sFlt-1 or PlGF with lipoproteins and exosomes. A**, Cholesterol, sFlt-1, free PlGF, and total PlGF (mean±SEM) in fast-protein liquid chromatography (FPLC; Pharmacia 1983) fractions, obtained by applying serum samples of 6 nonpreeclamptic women and 11 preeclamptic women to a FPLC system (Pharmacia 1983) involving a Tricorn Superdex 200 10-300 GL and a Tricorn Superose 6 10-300 GL size exclusion column in series (GE Healthcare). Cholesterol was measured with a Selectra E Analyzer (DDS Diagnostic System, Istanbul, Turkey), and sFlt-1 and PlGF by commercial ELISAs (R&D Systems; DY321B and DY264). Total PlGF was determined by placing the samples in a heating block at 70 °C for 10 minutes before the measurement. **B**, Western blot of apolipoprotein A-I (ApoA-I), apolipoprotein B (ApoB), and the exosomal marker CD9 in the aforementioned FPLC fractions, making use of primary antibodies directed against ApoA-I (Proteintech, 14427-1-AP, 1:1000 dilution), ApoB (Proteintech, 20578-1-AP, 1:1000 dilution), and CD9 (Invitrogen, 13-0098-82, 1:1000 dilution). **C**, Pull-down of anti-PlGF (2 μg/mL; R&D Systems) or anti-Flt-1 (2 μg/mL; R&D Systems), added to a serum pool for 30 minutes at room temperature, by Dynabeads Streptavidin (Thermo Fisher), followed by immunoblotting of PlGF, sFlt-1, CD9, ApoA-I, and ApoB. **D**, Serum sFlt-1 and PlGF levels in a serum pool (mean±SEM of n=3), without or with preincubation for 30 minutes at room temperature with 5 μg/mL anti-PlGF, 5 μg/mL anti-Flt-1, or a mixture of anti-CD9 (5 μg/mL; Invitrogen), anti-ApoA-I (5 μg/mL; Thermo Fisher), anti-ApoB (5 μg/mL; R&D Systems), and then pulled down by Dynabeads Streptavidin (Thermo Fisher). **E**, sFlt-1 and PlGF (mean±SEM) in FPLC fractions obtained by applying placental perfusate samples of 4 healthy placentas to a FPLC system. **P<0.01, ***P<0.001, and ****P<0.0001 vs control. CM indicates chylomicrons; HDL, high-density lipoprotein; IDL, intermediate-density lipoprotein; LDL, low-density lipoprotein; PlGF, placental growth factor; sFlt-1, soluble Fms-like tyrosine kinase-1; and VLDL, very-low-density lipoprotein.

As expected, ApoA-I (apolipoprotein A-I) was predominantly found in the HDL2- and HDL3-containing fractions (Figure [B]), while ApoB (apolipoprotein B) was confined to the LDL-containing fraction (Figure [B]). Exosomal marker CD9 occurred in the LDL-, HDL2-, and HDL3-containing fractions (Figure [B]). These data imply that PlGF and sFlt-1 occurring in the LDL-, HDL2-, and HDL3-containing fractions might either be lipoprotein-bound or exosomal.

Next, we pulled down endogenous PlGF and sFlt-1, making use of PlGF and sFlt-1 antibodies added to the abovementioned serum pool, and again observed the coprecipitation of PlGF and sFlt-1 with CD9, ApoA-I, and ApoB (Figure [C]). Moreover, when remeasuring serum sFlt-1 and PlGF in the serum pool after removing CD9, ApoA-I, and ApoB with an antibody pull-down, the levels of both sFlt-1 and PlGF were highly reduced (Figure [D]). FPLC revealed that the latter reduction particularly occurred in the HDL3-containing fraction (data not shown). These findings confirm that circulating PlGF occurs exclusively in either lipoproteins or exosomes, while sFlt-1 seems to be additionally present in an unbound form.

Finally, given the predominant placental origin of both sFlt-1 and PlGF,^5^ we applied FPLC to the maternal perfusate of 4 healthy human term placentas perfused with buffer in a closed-circuit setup for 3 hours. Under this condition, sFlt-1 was present in the LDL- and HDL2-containing fractions, while PlGF occurred in the LDL-, HDL3-, and free protein-containing fractions (Figure [E]). Given the absence of lipoprotein synthesis in the placenta, these data support the concept of placental release of exosomes containing sFlt-1 and PlGF. Although the placental release of unbound (nonexosomal) sFlt-1 and PlGF is likely, such release appeared to be minor as the levels of both proteins in the free protein-containing fraction were close to or below the detection limit.

Taken together, our experiments are the first to demonstrate that both sFlt-1 and PlGF associate with LDL, HDL, and exosomes. We provide 4 lines of evidence to support this conclusion: (1) FPLC of serum from pregnant women demonstrating sFlt-1 and PlGF in the LDL and HDL fractions; (2) immunocapture of endogenous sFlt-1 or PlGF-associated lipoproteins and exosomes; (3) the reduction of serum sFlt-1 or PlGF after LDL, HDL, and exosome pull-down; and (4) the placental release of sFlt-1 and PlGF in exosomes. Our data would imply that nonexosomal PlGF, released from either the placenta or nonplacental sites, rapidly binds to lipoproteins. Although this is also true for sFlt-1, serum sFlt-1, unlike PlGF, additionally occurred independently of LDL, HDL, and exosomes, that is, unbound. In the compartments where sFlt-1 and PlGF occur together, the majority of PlGF is sFlt-1-bound and could only be demonstrated after sFlt-1 destruction by heating. Preeclampsia particularly lowered the levels of free PlGF by increasing the sFlt-1 content of the HDL2 and HDL3 fractions. Apheresis likely improves the [sFlt-1]/[free PlGF] ratio by depleting the LDL fraction but would not affect sFlt-1 in the HDL fraction. Future studies are required to unravel what controls the PlGF and sFlt-1 content of lipoproteins and exosomes and to what degree this is altered in preeclampsia. Ultimately, this may help to improve the diagnosis of preeclampsia and restore the angiogenic imbalance in this disorder.

## ARTICLE INFORMATION

### Sources of Funding

L. Tan and K. Verdonk are supported by the Stichting Lijf en Leven.

### Disclosures

None.
